# There is no relationship between Paraoxonase serum level activity in women with endometriosis and the stage of the disease: an observational study

**DOI:** 10.1186/1742-4755-10-32

**Published:** 2013-06-22

**Authors:** Felipe Barca Bragatto, Caio Parente Barbosa, Denise Maria Christofolini, Carla Peluso, Aline Amaro dos Santos, Fernanda Abani Mafra, Viviane Cavalcanti, Sonia Hix, Bianca Bianco

**Affiliations:** 1 Division of Biochemistry, Department of Morphology, Faculdade de Medicina do ABC, Santo André, Santo André/SP, Brazil; 2 Human Reproduction and Genetics Center, Department of Gynecology and Obstetrics, Faculdade de Medicina do ABC, Av. Principe de Gales, Santo André/SP, 821, Brazil

**Keywords:** Endometriosis, Paraoxonase, Oxidative stress, Infertility, High-density lipoprotein

## Abstract

**Background:**

Endometriosis is a chronic condition whose pathophysiology is unknown, but there is evidence suggesting a link with oxidative stress. Paraoxonase is a serum enzyme which circulates associated with high-density lipoprotein (HDL). It acts protecting HDL and LDL of lipid peroxidation. We aimed to compare the serum levels of PON-1 activity in women with endometriosis in different stages of the disease (minimal/mild and moderate/severe).

**Methods:**

80 infertile women with endometriosis diagnosed by laparoscopy/laparotomy with histologic confirmation of the disease were divided according to the American Society for Reproductive Medicine classification in minimal/mild (n = 33) and moderate/severe (n = 47) cases. Paraoxonase activity and arilesterase activity were measured by spectrophotometry. Body mass index and fasting glucose levels were also determined.

**Results:**

The paraoxonase activity were 191.29 ± 22.41 U/l in women with minimal/mild endometriosis and 224.85 ± 21.50 U/l in women with moderate/severe disease (*P* = 0.274). Considering arilesterase level, the results showed 89.82 ± 4.61 U/l in women with minimal/mild endometriosis and 90.78 ± 3.43 U/l in moderate/severe disease (*P* = 0.888).

**Conclusions:**

Evidence of lower paraoxonase activity in women with endometriosis was not found in this study. Besides, no difference was found considering minimal/mild or moderate/severe endometriosis.

## Background

Endometriosis is a chronic condition characterized by tissue histologically similar to the endometrium implants, grows and develops outside the uterine cavity associated with pelvic pain and infertility [1,2]. It affects 3-10% of women in their reproductive years and 20-50% of women with infertility [3]. The pathophysiologic mechanism is unknown, but some authors suggest a link with oxidative stress [4-9]. In the presence of pelvic endometriosis, peritoneal macrophages would be activated, increasing the production of reactive oxygen species (ROS) [4], one of the responsible for the inflammatory reaction observed in endometriosis [6]. Increased levels of lipid peroxidation markers in peritoneal fluids of women with endometriosis [10], like tumor necrosis factor (TNF) α, were found in previous published studies [9].

Those markers are chemotactic factors for monocytes and T-lymphocytes, such the T-helper, which immune response has been identified as a main factor in the development and progression of endometriosis [5]. An increase in the oxidation of low-density lipoprotein (LDL) was reported in endometriosis patients [8].

Paraoxonase1 (PON1) is an antioxidant serum enzyme which circulates associated with HDL (High Density Lipoprotein) [11-13]. It hydrolyzes various organophosphate compounds, including pesticides and neurotoxic gases. Its name derives from paraoxon, a metabolite of an ordinary pesticide called parathion, which is hydrolyzed by PON1 with modest catalytic efficiency [13]. The PON1 has an appreciable arylesterase activity, being phenylacetate the typical substrate. Khersonsky et al. [14] suggests that PON1 is in fact,a lactonase and not an esterase or a fosfotriesterase as traditionally described.

PON1 is also involved in the metabolism of drugs, being used for their inactivation [15]. The identity of the native substrate of PON1 and other PON’s family enzymes is not known yet. A possible physiological substrate is homocysteine-thiolactone, which may be formed from the non-protein amino acid homocysteine. Serum levels of homocysteine are known as a risk factor for atherosclerotic vascular disease by inducing endothelial injury. One of the potential mechanisms that explain homocysteine’s toxic effects is homocysteine-thiolactone’s formation, a metabolite that can modify proteins, including the LDL apoprotein B and HDL Apo A, favoring atherogenesis [16].

Paraoxonase features two genetic polymorphisms that results in different paraoxonase activity against the substrate paroxon, particularly when the test is performed in the presence of NaCl (paraoxonase activity), but not against the substrate phenylacetate (arilesterase activity) [12]. Most studies that have attempted to relate the PON_192_ polymorphism with diseases, particularly cardiovascular disease, were inconclusive.

This enzyme acts protecting HDL and LDL of lipid peroxidation, degrading cholesterol esters’ oxidized lipids and phospholipids present in the lipoproteins, erythrocytes and macrophages. PON1 may otherwise be inactivated by oxidized lipids [17]. Furthermore, PON1 has additional antiatherogenic properties, as inhibition of cholesterol biosynthesis by macrophages and stimulating macrophage cholesterol efflux [18-21]. Paraoxonase activity measurements have shown an inverse association with various morbidities, like obesity, cardiovascular disease, diabetes, metabolic syndrome, and aging, all related with its antioxidant activity [22-25].

Considering the complex cellular and molecular mechanisms involved in endometriosis formation and progression, we aimed to compare the serum levels of PON-1 activity in women with endometriosis in different stages of the disease (minimal/mild and moderate/severe).

## Methods

### Patients

The studied group consisted of 80 infertile women with endometriosis attended at Human Reproduction and Genetics Center of the Faculdade de Medicina do ABC, Santo André, Brazil. Patients with endometriosis-associated infertility were diagnosed by laparoscopy and classified according to the American Society for Reproductive Medicine [26], with histologic confirmation of the disease. In this group, minimal/mild (stage I and II) endometriosis was found in 33 cases (41.2%) and moderate/severe (stage III and IV) endometriosis in 47 cases (58.8%).

The cause of infertility was investigated including a hormonal and biochemical profile, testing for sexually transmitted diseases, imaging examinations, investigation of genetic and/or immunological abnormalities, hysterosalpingography, hysteroscopy and laparoscopy (performed in all women up to 36 years of age and also in patients over 36, whenever there were symptoms or abnormalities on the imaging examinations), and semen analysis of the partner. Women with endometriosis who did not achieve pregnancy after at least twelve natural or induced cycles following laparoscopy were considered infertile. Patients with obesity (BMI ≥ 30 kg/m^2^), smokers and with acute or chronic medical conditions and whose partner had any male factors associated with infertility were excluded from the study.

Clinical data and peripheral blood samples were collected only after explaining the objectives of the study and obtaining signed informed consent, as approved by the local Research Ethics Committee.

### Laboratory evaluation

5 mL of blood were collected by trained professionals through a peripheral vein, in a room protected from direct light. PON activity was determined in blood samples. The serum was isolated by centrifugation at low speed (5.000 rpm for 3 minutes) after coagulation. The samples were stored at -80°C. Paraoxonase activity and arilesterase activity were determined spectrophotometrically using paraoxon and phenylacetate as substrates, respectively.

The paraoxonase activity was determined using a PON paraoxon (diethyl - p-nitrophenyl) as substrate and measuring the increase in absorbance at 412 nm by the formation of 4 - nitrophenol. The activity was measured at 30°C during 90 seconds after 12 μl of serum were added to each well containing 238 μl of Tris/HCl (100 mmol/l, pH 8.0) buffer containing 2 mmol/l CaCl_2_ and 5.5 mmol /l of paraoxon. The regeneration rate of 4-nitrophenol was measured at 412 nm in a visible plaque spectrophotometer. At the same time the spontaneous hydrolysis of paraoxon was discounted in a reference well containing only the buffer with the reagent. The paraoxonase activity of PON1 was determined in quintuplicate and all results are expressed in U/ml. The enzyme activity was calculated by the molar extinction coefficient of 17.100 M^-1^. cm^-1,13^ One unit of paraoxonase activity is defined as 1 nmol of 4-nitrophenol formed per minute. Alternatively paraoxonase activity was measured in buffer containing 1 M NaCl^13^ in order to verify the polymorphism increased activity.

PON1’s arilesterase activity in serum was determined spectrophotometrically by using the synthetic substrate phenylacetate. The reaction was started by adding 23 ul of phenylacetate (10 mM) to wells containing 5 ul of serum (prediluted 1:13) and 222 uL of buffer 50 mmol/l Tris pH 8.0/1 mmol CaCl_2._ The production of phenol was determined spectrophotometrically during 1 minute at 270 nm, using a molar extinction coefficient of 1310/mol/cm at pH 8.0. The spontaneous hydrolysis of paraoxon was discounted measuring the absorbance in a well containg only the buffer and phenylacetate. The PON1’s arilesterase activity was determined in quintuplicate and all results were expressed as U/ml. 1U of phenylacetate hydrolyses 1 μmol arilesterase per minute [22-24].

BMI was calculated according to the Quetelet formula, by dividing the weight in kilogram by the squared height in metres (kg/m2). The blood glucose level was determined using commercially available assay kits. The patients were fasting for at least 8 hours before their blood were collected for laboratory glucose level evaluation.

### Statistical analyses

Statistical analyses were made using Minitab 16 for Windows [27]. We assumed a significance level of 0.05 for the statistical tests. Normality of data distribution of women with minimal/mild and moderate/severe endometriosis for both enzymes were verified using the Anderson-Darling normality test. Differences among the minimal/mild and moderate/severe endometriosis for PON1’s paraoxonase and arilesterase activity were assessed using the Mann–Whitney U test. Correlation between PON1’s paraoxonase and arilesterase activity and clinical severity of the disease was performed by Sperman’s rank test.

## Results

The group mean age was 33.5 ± 7.5 years, with a mean body mass index (BMI) of 24.02 ± 5.88 kg/m^2^. The mean fasting glucose levels were 93 ± 26 mg/dl.

The serum PON1’s paraoxonase and arilesterase activities in each group are presented in Table 1. We did not find any difference in women with minimal/mild and moderate/severe endometriosis for both enzymes, even when the patients were analyzed separately according to endometriosis stage (I, II, III and IV endometriosis).

**Table 1:** Serum PON1’s paraoxonase and arilesterase activities in women with minimal/mild and moderate/severe endometriosis ± (standard deviation) and P significance level

	**Minimal/mild endometriosis**	**Moderate/severe endometriosis**
PON1’s paraoxonase activity (U/l)	191.29 ± 22.41^a^	224.85 ± 21.50
PON1’s arilesterase activity (U/l)	89.82 ± 4.61^b^	90.78 ± 3.43

^*a*^*P* = 0.274 vs women with Moderate/severe endometriosis.

^*b*^*P* = 0.888 vs women with Moderate/severe endometriosis.

## Discussion

In the present study, we hypothesized a possible relation between paraoxonase activity and endometriosis development. However, we found no difference between the PON1’s paraoxonase and arilesterase activity comparing the group with minimal/mild stage disease with the one with moderate/severe endometriosis. Besides, there was no correlation with the stage of the disease and PON1’s activity. Analysis considering the four stages separately was performed, but it also did not showed positive correlation.

Verit et al. [10] have supposed, previously, the association of PON1’s activity and endometriosis. The authors studied 87 women who underwent laparoscopy or laparotomy (40 control patients with no pathologic findings; 24 women with minimal/mild endometriosis and 23 women with moderate/severe endometriosis). PON-1 activity was significantly higher in women with moderate/severe endometriosis than in women with minimal/mild disease and controls, and in women with minimal/mild endometriosis compared with control groups (P < 0.0001, for all). A significant negative correlation was found between PON-1 activity and stage of the disease (r520.74, P < 0.0001). The authors conclude that PON-1 activity can be used as a diagnostic test to detect endometriosis.

Although we didn’t have a control group, the average activity of the enzyme we found in this study was not very different from the activity found by other studies in women without endometriosis (183.7 ± 22.3 U/l in 40 women with no endometriosis in the study of Verit et al., 2008 [10]; 258.29 ± 101.95 U/l in 22 adult females with no endocrine or metabolic disorders or history of drug use in the study of Ozenoglu et al., 2008 [28]; and 240 ± 144 U/l in 200 individual, both men and women, from blood bank serum samples in a study performed by Davis et al., 2009 [29]). Besides, in our study the patients were diagnosed with endometriosis by laparoscopy/laparotomy, and classified according to the American Society for Reproductive Medicine [26] with histological confirmation of the disease, which gives a more accurate diagnosis of the stage of the endometriosis in comparison with visual confirmation alone. Barbosa et al. (2009) [2] observed endometriosis in healthy peritoneum biopsy of fertile and asymptomatic women, showing the importance of histological confirmation for minimal/mild endometriosis.

Studies involving PON1 have attributed an antioxidant and ateroprotector role to it. They have demonstrated that reduced paraoxonase activity are observed in adults with various diseases such as diabetes mellitus, renal failure, obesity, and cardiovascular disease [24,25,30-32]. Others have shown an increased activity of PON1 in individuals who maintains a physical activity routine, which is a protective factor against cardiovascular disease by improving the quality of their HDL [11,33]. However, there is no clear evidence that women with endometriosis have more atherosclerosis than the general population [34,35].

Despite the evidence that oxidative stress may play a role in endometriosis [4-9], there are other theories that may explain the pathophysiology of this disease. The implantation theory proposes that the retrograde menstruation may result in implantation and growth of endometrial cavity. The coelomic metaplasia suggests that there’s a transformation of peritoneal in mullerian cells, activated by hormones. Combining the two previous theories, the induction theory attributes to immunological and endogenous biochemical factors the induction of the differentiation of undifferentiated cells into endometrial tissue. Finally, recent studies have shown genetic predisposition and familial tendency of endometriosis. None of these theories alone have succeeded to determine the mechanism of the disease [25].

Some authors have also suggested the possibility of endometriosis is a disease caused or associated with oxidative stress [36-40]. In the presence of pelvic endometriosis, there was activation of macrophages in the peritoneal cavity, which could promote increased production of reactive oxygen species and nitrogen and, consequently, oxidative stress, resulting in lipid peroxidation, its degradation products and the products formed by its interaction with low-density lipoproteins and other proteins [41]. Oxidized lipids, to decompose, generate products such as malondialdehyde and could be recognized as foreign, antigenic response with consequent triggering antibody production. This process leads to oxidative damage to red blood cells, and peritoneal endometrial cells which, in turn, stimulate the recruitment and activation of mononuclear phagocytes further perpetuating oxidative damage in the pelvic cavity. Oxidative stress also damages mesothelial cells and can induce the appearance of adhesion sites for endometrial cells, favoring the development and progression of endometriosis [40,42].

Although there is evidence suggesting the presence of oxidative stress in the sites of pelvic endometrial implants, little is known about the systemic oxidative status in women with infertility related to endometriosis. Likewise, very little is known about the association between disease stage and systemic markers of oxidative stress [40].

The human biological aging is a phenomenon that is associated with changes in the activity of cells, tissues and organs, as well as reducing the effectiveness of a set of processes physiological. With respect to aspects of the endogenous production of oxidants, both the causes and the molecular mechanisms involved this process is not yet sufficiently known. However, evidences attributed to aging palatine accumulation of structural and functional changes in macromolecules and cell membranes, caused by the deleterious effects of free radicals and other reactive oxygen species which form spontaneously in the mitochondria as a consequence of oxidative metabolism [43]. Thus, one of the possible causes of no association between PON activity and endometriosis can also be attributed to the younger age of the patients.

Furthermore, PON’s activity varies widely between individuals, partly related to polymorphisms present in population [23,44,45] and to serum HDL’s level, as HDL is pointed as the responsible to distribute PON to the tissues, where it could exercise its antioxidant function [46-49].

Because of those difficulties in comparing PON’s activity between individuals, we suggest that perhaps a better way of determining the influence of this enzyme in endometriosis in further studies is correlating the same individual PON’s activity along the time, with the evolution of the disease and response to treatments, corrected by the patient HDL levels.

In conclusion, evidence of lower paraoxonase activity in women with endometriosis was not found in the present study. Besides, no difference was found considering minimal/mild or moderate/severe endometriosis.

## Competing interests

The authors have no competing interests.

## Authors’ contributions

FBB, SH and BB conceived study design. FBB, CP, AAdosS, FAM and VC performed the data collection and analysed data. SH, BB, DC and CPB interpretation the data. All authors were involved in literature search, writing the paper and had final approval of the submitted and published versions. All authors read and approved the final manuscript.
